# Cytochrome C Oxidase Subunit 4 (COX4): A Potential Therapeutic Target for the Treatment of Medullary Thyroid Cancer

**DOI:** 10.3390/cancers12092548

**Published:** 2020-09-08

**Authors:** Athanasios Bikas, Kirk Jensen, Aneeta Patel, John Costello, Sarah M. Reynolds, Maria Cecilia Mendonca-Torres, Shilpa Thakur, Joanna Klubo-Gwiezdzinska, Dorina Ylli, Leonard Wartofsky, Kenneth Burman, Vasyl Vasko

**Affiliations:** 1 MedStar Health Research Institute, Georgetown University Hospital and Washington Hospital Center, 110 Irving St NW, Washington, DC 20010, USA; dorina.ylli@umed.edu.al (D.Y.); leonard.wartofsky@medstar.net (L.W.); Kenneth.D.Burman@medstar.net (K.B.); 2 Department of Pediatrics, Uniformed Services University of the Health Sciences, 4301 Jones Bridge Road, Bethesda, MD 20814, USA; kirk.jensen@usuhs.edu (K.J.); aneeta.patel@usuhs.edu (A.P.); john.costello.ctr@usuhs.edu (J.C.); sarah.m.reynolds6.mil@mail.mil (S.M.R.); maria.mendonca-torres@usuhs.edu (M.C.M.-T.); vasyl.vasko.ctr@usuhs.edu (V.V.); 3 National Institute of Diabetes and Digestive and Kidney Diseases, National Institutes of Health, Bethesda, MD 20814, USA; shilpa.thakur@nih.gov (S.T.); joanna.klubo-gwiezdzinska@nih.gov (J.K.-G.)

**Keywords:** thyroid cancer, medullary thyroid cancer, COX4, cytochrome-c oxidase, cancer metabolism

## Abstract

**Simple Summary:**

Targeting the metabolism of cancers is a promising strategy for treatment. In the current study we examine the role of COX4, an enzyme of the mitochondria, in thyroid cancer. The experiments were performed in immortalized cell lines and tissue sample from human thyroid cancers. We examined several types of thyroid cancers, and we found out that the role of COX4 was more pronounced in medullary thyroid cancer. As a result, we believe that COX4 should be studied further as a therapeutic target in medullary thyroid cancer.

**Abstract:**

The nuclear-encoded subunit 4 of cytochrome c oxidase (COX4) plays a role in regulation of oxidative phosphorylation and contributes to cancer progression. We sought to determine the role of COX4 in differentiated (DTC) and medullary (MTC) thyroid cancers. We examined the expression of COX4 in human thyroid tumors by immunostaining and used shRNA-mediated knockdown of COX4 to evaluate its functional contributions in thyroid cancer cell lines. In human thyroid tissue, the expression of COX4 was higher in cancers than in either normal thyroid (*p* = 0.0001) or adenomas (*p* = 0.001). The level of COX4 expression correlated with tumor size (*p* = 0.04) and lymph-node metastases (*p* = 0.024) in patients with MTCs. COX4 silencing had no effects on cell signaling activation and mitochondrial respiration in DTC cell lines (FTC133 and BCPAP). In MTC-derived TT cells, COX4 silencing inhibited p70S6K/pS6 and p-ERK signaling, and was associated with decreased oxygen consumption and ATP production. Treatment with potassium cyanide had minimal effects on FTC133 and BCPAP, but inhibited mitochondrial respiration and induced apoptosis in MTC-derived TT cells. Our data demonstrated that metastatic MTCs are characterized by increased expression of COX4, and MTC-derived TT cells are vulnerable to COX4 silencing. These data suggest that COX4 can be considered as a novel molecular target for the treatment of MTC.

## 1. Introduction

Metabolic reprogramming of tumor cells is being increasingly recognized as an important disease driver, controlling various aspects of malignant progression [1]. Mutations in genes involved in mitochondrial metabolism activate the stress response pathway and therefore contribute to tumorigenesis [2]. The increased production of mitochondrial reactive oxygen species (ROS) and changes in the cellular redox status alter the activity of transcription factors that stimulate cancer cell proliferation [3]. Mitochondrial dysfunction and aberrant expression of molecules controlling energy metabolism have been reported in various types of cancers, including gliomas [4], prostate [5] and breast cancer [6].

The adaptation of tumor cell energy metabolism was suggested to be a key element underlying cancer cell survival in metastatic sites [6]. The functional activity of the mitochondrial electron transport chain (ETC) molecules plays a crucial role in this process. ETC is the site of oxidative phosphorylation, which provides energy to power ATP synthase. ETC is also a major site of premature electron leakage to oxygen, generating superoxide and potentially resulting in increased oxidative stress. The terminal complex of the ETC, cytochrome c oxidase (COX), catalyzes the transfer of electrons from cytochrome c to molecular oxygen and is believed to be the primary regulatory site for oxidative phosphorylation [7].

COX is a bigenomic enzyme with 10 of the 13 subunits being encoded by nuclear DNA and the remaining 3 by mitochondrial DNA. The largest of the nuclear encoded subunits of the COX complex, COX4, is known to function as a regulatory subunit, inhibiting the enzyme activity at high adenosine triphosphate (ATP) concentrations [8]. There are two COX4 isoforms (COX4-1 and COX4-2), and their expression is regulated by O_2_. Under conditions of reduced O_2_ availability, hypoxia-inducible factor 1 (HIF-1α) reciprocally regulates COX4 subunit expression by activating transcription of the genes encoding COX4-2 and LON protease, a mitochondrial protease that is required for COX4-1 degradation [9]. It has been shown that the allosteric inhibition of the COX complex occurs by binding of ATP to COX4 [10]. These properties of COX4 place it in a pivotal physiological position to regulate cellular energy metabolism, and optimize the efficiency of respiration at different concentrations of O_2_.

The pharmacological targeting of mitochondrial functions has emerged as a promising tool for treatment of cancer. Metformin, a mitochondrial complex I inhibitor, inhibited growth and downregulated the mTOR/p70S6K/pS6 signaling pathway in thyroid cancer cell lines [11,12]. In an animal model of thyroid cancer, mitochondrial glycerophosphate dehydrogenase (mGPDH) was identified as a molecular target for metformin, and downregulation of mGPDH by metformin led to inhibition of thyroid cancer growth in vivo [13]. In medullary thyroid cancer (MTC) cells, treatment with metformin was associated with downregulation of MAPK/ERK signaling and decreased resistance to anoikis [14]. The mitochondria-targeted redox-sensitive agent, Mito-CP, suppressed growth of cancer cells in an animal model of medullary thyroid cancer. Treatment with Mito-CP led to mitochondrial membrane depolarization, decreased oxygen consumption, and increased oxidative stress in medullary thyroid cancer cells [15].

Together these observations formed a rationale to further explore the role of mitochondria in thyroid cancer. In this study, we focused on the role of the well characterized regulator of oxidative phosphorylation COX4 in thyroid cancer. We examined the expression of COX4 in a series of human thyroid cancers and in thyroid cancer cell lines. To clarify the functional implication of COX4 in thyroid cancer cells, we performed silencing experiments, and examined thyroid cancer cell response to treatment with pharmacological inhibitors of COX4.

## 2. Results

### 2.1. COX4 Expression Is Increased in Differentiated Thyroid Cancers

**Figure 1:**
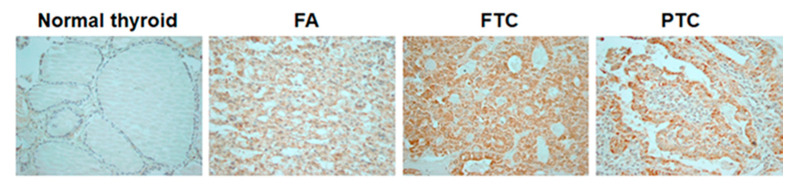
COX4 expression in human thyroid tissue samples. Immunostaining with anti-COX4 indicating minimal expression of COX4 in normal tissue samples, compared with the moderate expression in follicular adenoma (FA), and strong, homogeneous cytoplasmic expression in follicular thyroid cancer (FTC) and papillary thyroid cancer (PTC) (magnification ×400).

In normal thyroid tissue, immunostaining with a COX4-specific antibody was positive in 1/48 (2.1%) examined samples. In follicular-cell derived thyroid tumors, positive staining with anti-COX4 was detected in 1/14 (7.1%) FAs, 11/26 (42.3%) FTCs and 42/76 (55.3%) PTCs. The positive staining was more frequently detected in thyroid tumors than in normal thyroid tissue (Figure 1).

The results of COX4 immunostaining in normal thyroid tissue, benign and malignant thyroid lesions are summarized in Table 1.

The level of COX4 expression was not significantly different between PTCs and FTCs. No significant associations were found between COX4 expression and patients’ gender, age, tumor size and presence of lymph node metastases at the time of surgery.

To determine a possible association between the level of COX4 expression and thyroid oncogene mutations, we analyzed *BRAFV600E* in nucleic acids extracted from 20 PTCs. Nine out of 20 examined PTCs harbored *BRAFV600E*. The levels of COX4 expression were not significantly associated with presence of *BRAFV600E* in PTCs. Positive staining with anti-COX4 was detected in 5/9 *BRAFV600E*-positive PTCs, and in 7/11 *BRAFV600E*-negative PTCs.

### 2.2. RET-Positive MTCs Demonstrate a High Level of COX4 Expression

Expression of COX4 was examined by immunohistochemistry in 65 patients with MTC and the results of immunostaining are summarized in Table 2.

Analysis of COX4 in MTCs revealed positive staining in 49/65 (75.3%) cases. There were no associations between the level of COX4 expression and patient’s age or gender. An association was found between COX4 and tumor size (*p* = 0.04) (median (IQR) was 1.5 cm (1–2 cm) vs. 2 cm (1.2–3.8 cm) respectively for COX4 negative and COX4 positive tissue). Furthermore, COX4 was significantly associated with the presence of lymph-node metastasis at the time of surgery. Positive immunostaining with anti-COX4 was detected in 25/28 (89.3%) MTCs presenting with lymph nodes metastases versus 24/37 (64.9%) tumors without metastases (*p* = 0.024).

DNA samples from 41 MTCs were subjected to genomic analysis. There were 22 cases with *RET* mutations, 6 cases with *RAS* mutations and in 13 MTCs mutations were not detected.

**Figure 2:**
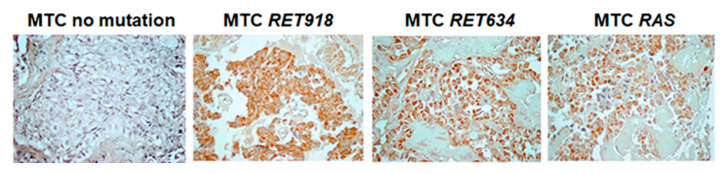
COX4 expression in human medullary thyroid tissue samples. Immunostaining with anti-COX4 indicating minimal expression of COX4 in MTC without detectable *RET* and *RAS* mutations. Strong, homogeneous cytoplasmic expression in MTC samples harboring *RET* and *H2RAS* mutations (magnification, ×400).

Positive staining with anti-COX4 was detected in 20/22 (90.9%) MTCs with *RET* mutations, 4/6 (66.6%) MTCs with *RAS* mutations and in 6/13 (46.1%) MTCs without detectable mutations. The level of COX4 immunostaining in MTCs with *RET* mutations was not significantly different from MTCs harboring *RAS* mutations but was significantly higher as compared to MTCs without detectable mutations (*p* = 0.006). The patterns of immunostaining with anti-COX4 in MTC tissue samples are presented in Figure 2.

### 2.3. Analysis of COX4 Protein and mRNA in Thyroid Cancer Cell Lines

**Figure 3:**
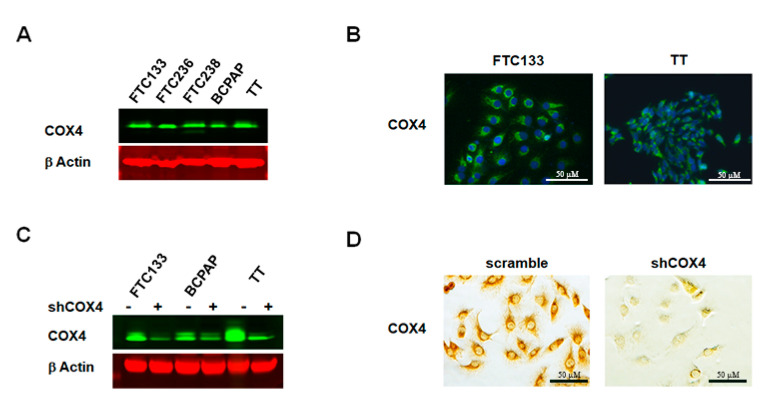
COX4 expression and efficiency of COX4 silencing in thyroid cancer cell lines. (**A**) Western blot analysis shows expression of COX4 in thyroid cancer cells with no significant differences between FTC-, PTC-, and MTC-derived cells. (**B**) Immunostaining with COX4 (green) and DAPI (blue) in FTC133 and TT cells demonstrates perinuclear expression of COX4. (**C**) Western blot using protein lysate of thyroid cancer cells transfected with scramble or COX4 specific shRNA demonstrates downregulation of COX4 in cells transfected with shCOX4. (**D**) Loss of COX4 immunostaining in FTC133 cells after transfection with shCOX4.

We next assessed the expression of COX4 in thyroid cancer cell lines that derived from FTCs (FTC133, FTC236 and FTC238), PTC (BCPAP), and MTC (TT). Western blot with anti-COX4 demonstrated expression of COX4 protein (Figure 3A) in all examined cell lines. Results of immunostaining were consistent with Western blot data and cytoplasmic expression of COX4 was detected in all thyroid cancer cells. Figure 3B demonstrates predominantly perinuclear staining, which is consistent with mitochondrial COX4 expression.

Since the anti-COX4 antibody that was used in this study is not specific for COX4 isoforms, we next examined COX4 isoform 1 and COX4 isoform 2 by RT PCR using isoform-specific primers. Analysis of the COX4 mRNA levels showed that isoform 1 but not isoform 2 was predominantly expressed in all examined thyroid cancer cells lines.

To clarify the role of COX4 in thyroid cancer cells, we generated COX4-deficient FTC133, COX4-deficient BCPAP and COX4-deficient TT cells using lentiviral transfection with COX4 isoform 1-specific shRNAs. After transfection with shCOX4, the mRNA level of COX4 isoform 1 was decreased to 0.2 fold in FTC133 cells, 0.1 fold in BCPAP cells, and 0.05 fold in TT cells. The inhibition of COX4 expression was confirmed at the protein level by Western blot (Figure 3C), and by immunostaining (Figure 3D).

Silencing of COX4 isoform 1 was not associated with compensatory overexpression of COX4 isoform 2. The downregulation of COX4 was not associated with changes in expression of mitochondrial respiratory chain molecules. The protein levels of NDUF, SDHA, Cytochrome C and ATP5B were not significantly different in COX4-expressing and COX4-deficient cells.

### 2.4. Assessment of Mitochondrial Function in COX4-Expressing and COX4-Deficient Thyroid Cancer Cells

**Figure 4:**
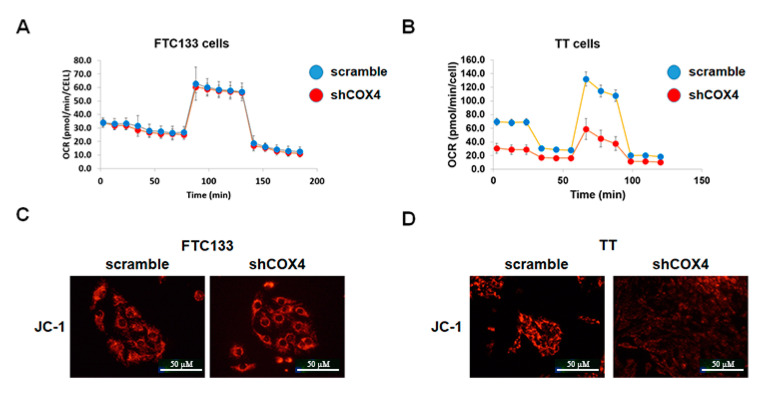
Assessment of mitochondrial function in COX4-expressing and COX4-deficient thyroid cancer cells. (**A**) Minimal effects of COX4 knockdown on mitochondrial respiration, ATP production, maximal respiration and spare respiratory capacity in FTC133 documented by Seahorse Mitostress Kit Assay (OCR—oxygen consumption rate pmol/min/cell). (**B**) Inhibition of the baseline mitochondrial respiration and ATP production, in TT cells transfected with shCOX4 as compared to TT cells transfected with scramble vector documented by Seahorse Mitostress Kit Assay. (**C**) In FTC133, COX4 silencing had no effects on mitochondrial membrane potential as demonstrated by JC-1 staining. (**D**) In TT cells, JC-1 staining was decreased after silencing with shCOX indicating the role of COX4 in maintenance of mitochondrial membrane potential in this MTC-derived cell line.

We investigated the levels of mitochondrial oxidative phosphorylation in thyroid cancer cell lines using the XF Cell Mito Stress Test Kit. At baseline conditions, the oxygen consumption and mitochondrial ATP production were higher in MTC-derived TT cells as compared to FTC- and PTC-derived cell lines. COX4 silencing had no significant effects on oxygen consumption, and did not affect the levels of maximal respiration and spare respiratory capacity regulated by mitochondrial respiration modulators in the FTC-derived cell line (Figure 4A). Similar results were found in PTC-derived cells. In contrast, COX4-deficient TT cells demonstrated significantly lower oxygen consumption rates (OCR), and decreased mitochondrial ATP production as compared to COX4-expressing TT cells (Figure 4B).

We also performed JC-1 staining to examine the mitochondrial membrane potential in COX4-expressing and COX4-deficient thyroid cancer cells. As demonstrated in Figure 3C,D, the mitochondrial membrane potential was maintained in shCOX4 FTC133, but was decreased in shCOX4 TT cells.

### 2.5. The Effect of COX4 Silencing on Cell Signaling Activation in Thyroid Cancer Cells

The results of seahorse experiments demonstrating the effects of COX4 silencing on mitochondrial ATP production in TT cells prompted us to examine activation of AMP-inducible protein kinase (AMPK) signaling pathway in COX4-expressing and COX4-deficient cells. A previous study demonstrated that targeting mitochondria activates AMP-inducible protein kinase (AMPK), leading to inhibition of mTOR/p70S6K/pS6 signaling [16]. Therefore, we examined the phosphorylation status of AMPK/p70S6K/pS6 in control and COX4-deficient thyroid cancer cell lines.

**Figure 5:**
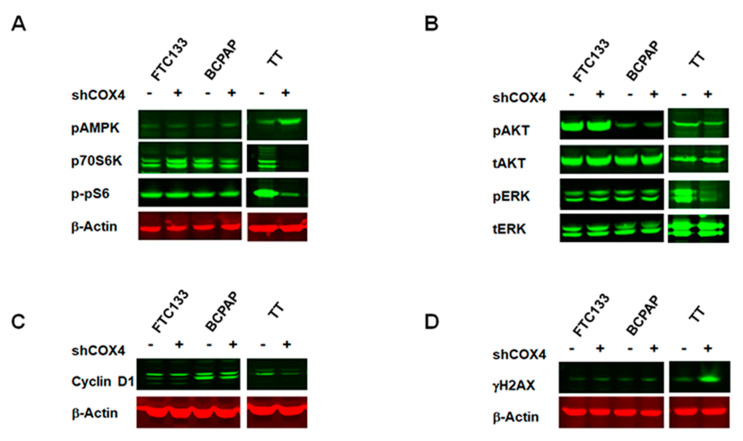
The effects of COX4 downregulation on cell signaling activation in thyroid cancer cells. (**A**) Western blot demonstrating cell type specific response to inhibition of COX4 with downregulation of p70S6K/p-pS6 in MTC-derived TT cells. (**B**) Inhibition of p-ERK in TT cells transfected with shCOX4. (**C**) Western blot demonstrating downregulation of cyclin D1 in shCOX4 TT cells. (**D**) Western blot with anti-γH2AX indicates activation of DNA-damage response in COX4 deficient TT cells.

As demonstrated in Figure 5A, the inhibition of COX4 was not associated with activation of AMPK in FTC133 as well as in BCPAP cells. The phosphorylation of p70S6K and pS6 was not significantly different between COX4-expressing and COX4-deficient cells.

In contrast, silencing of COX4 in MTC-derived TT cells was associated with activation of AMPK and downregulation of p70S6K and pS6. We also examined the effects of COX4 silencing on activation of ERK and AKT signaling pathways using phospho-specific antibody. The downregulation of COX4 in *PTEN*-deficient FTC133 cells had no effects on the level of p-AKT (Figure 5B). The inhibition of COX4 was not associated with changes in p-ERK in *BRAF*-positive BCPAP cells. In *RET634*-positive TT cells, COX4 silencing had a minimal effect on pAKT, but led to prominent inhibition of pERK.

### 2.6. The Effects of COX4 Silencing on Genes with an Established Role in Cancer

Since we showed COX4 silencing had inhibitory effects on p70S6K/pS6 and MAPK/ERK signaling pathways, we further sought to characterize the effects of COX4 silencing on expression of genes with an established role in carcinogenesis. We used RNA extracted from COX4-expressing and COX4-deficient FTC133, BCPAP and TT cells and performed real time PCR screening using the Human Cancer Pathway Finder RT^2^ Profiler PCR Array. Results of RT PCR screening are summarized in Table S1.

Downregulation of COX4 in FTC- and PTC-derived thyroid cancer cells had no significant effects on mRNA levels of genes controlling proliferation and apoptosis. In contrast, silencing of COX4 in MTC-derived TT cells was associated with downregulation of genes controlling cell cycle (*MKi67*, *MCM2*, *E2F4*, *SKP2* and *WEE1*), and up-regulation of genes related to DNA damage (*DDIT3*), senescence (*TBX2*), and hypoxia (*HMOX1* and *CA9*).

To confirm these observations, we performed Western blot analysis using protein lysate from COX4-expressing and COX4-deficient thyroid cancer cells. Western blot analysis demonstrated that the protein level of the cell cycle regulator cyclin D1 was not significantly affected by COX4 silencing in FTC133 as well as in BCPAP cells, but was decreased in MTC-derived TT cells (Figure 5C). Western blot with anti-γH2AX demonstrated that downregulation of COX4 had no effects in FTC- and PTC-derived cells, but induced activation of γH2AX in MTC-derived TT cells, suggesting activation of DNA damage inducible signaling (Figure 5D).

### 2.7. The Effects of Pharmacological Inhibitors of COX on Thyroid Cancer Cells

Results demonstrating the inhibitory effects of COX4 silencing on cell signaling activation in TT cells, prompted us to examine the effect of a pharmacological inhibitor of COX on thyroid cancer cells. For this purpose, we treated cells with potassium cyanide (KCN), a well-established inhibitor of COX activity.

**Figure 6:**
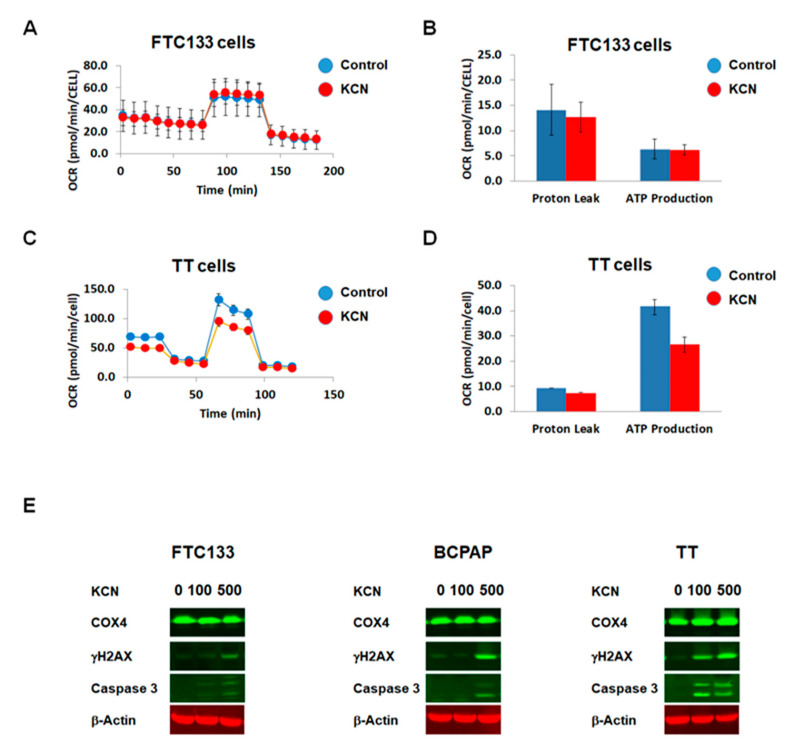
The effects of a pharmacological inhibitor of COX on thyroid cancer cells. (**A**) Seahorse Mitostress Assay demonstrates that treatment with KCN (100 μM for 24 h) had no effects on the oxygen consumption rate in FTC133 cells. (**B**) Treatment with KCN had no effects on ATP production in FTC133 cells. (**C**) Seahorse Mitostress Assay shows a decrease in oxygen consumption rate in TT cells that were treated with KCN (100 μM for 24 h). (**D**) The ATP production is lower in KCN-treated TT cells as compared to control. (**E**) Treatment with KCN induced DNA damage and caspase 3 cleavage in TT cells in dose dependent manner.

First we performed the seahorse assay and determined the effects of KCN on mitochondrial respiration. As demonstrated in Figure 6A,B, treatment with KCN at a concentration of 100 μM had no significant effect on the oxygen consumption rate and ATP production in FTC133 cells. Similar results were noted in BCPAP cells. However, treatment with KCN decreased the oxygen consumption rate and ATP production in TT cells (Figure 6C,D).

We also examined the effects of KCN on expression of COX4 as well as markers of DNA damage (γH2AX) and apoptosis (caspase 3 cleavage). In FTC133 and BCPAP cells, treatment with KCN at a concentration of 100 μM for 24 h was not associated with induction of DNA-damage-inducible signaling, and had no significant effects on caspase 3 cleavage (Figure 5D). Treatment with KCN at a concentration of 500 μM for 24 h had minimal effects on γH2AX and caspase 3 cleavage in FTC133. At this concentration KCN induced expression of γH2AX in BCPAP cells (Figure 6E). MTC-derived TT cells were more sensitive to KCN as compared to FTC133 and BCPAP cells. Treatment with KCN at a concentration of 100 μM for 24 h was associated with upregulation of γH2AX and caspase 3 cleavage, and these effects were even more prominent after treatment with KCN at a concentration of 500 μM.

Together these results demonstrated that MTC-derived cells are sensitive to treatment with a pharmaceutical inhibitor of COX activity, and suggested that COX4 could represent a therapeutic target in MTCs.

## 3. Discussion

Reprogramming metabolic fluxes is increasingly recognized as an important adaptation mechanism during tumor progression, controlling various aspects of development of malignancy [1,17]. Increased utilization of glucose for energy due to altered mitochondrial function is currently considered a hallmark of many proliferating tumors [18,19]. However, different cancer cell types undergo dissimilar bioenergetic alterations: some towards a more glycolytic state, while others favor the shuttling of fuels through oxidative phosphorylation [2]. Therefore, examination of tissue-specific mitochondrial characteristics is important in the understanding of metabolic changes that occur in cancer cells.

In our study, analysis of COX4 expression in a series of benign and malignant thyroid tumors showed a clear association between increased expression of COX4 and thyroid malignancy. In addition, cancer subtype specific patterns of COX4 expression were demonstrated, suggesting a role for oxidative phosphorylation in metastatic progression of MTCs, specifically in MTCs harboring *RET* mutations.

Cytochrome c oxidase is an important mitochondrial multi-protein complex and its role has been previously examined in various types of cancer. Several studies have provided data supporting the pro-carcinogenic role of COX. Indeed, it has been shown that COX is critically involved in establishing resistance to apoptosis in cervical cancer cells [20] and gliomas [21]. High COX activity was demonstrated in patients with high-grade gliomas and its activity was identified as an independent predictor of poor outcomes. Overexpression of a mitochondrial membrane markers, as well as of key components of COX, was demonstrated in breast cancer tissue samples [22]. A bioinformatics approach with data derived from breast cancer patients showed that transcriptional upregulation of mitochondrial oxidative phosphorylation genes in human breast tumors was specifically associated with metastasis [22].

However, a tumor suppressive role for COX was suggested in a study examining esophageal cancer cells [23]. It has been shown that disruption of cytochrome c oxidase function by COX4 silencing alters metabolic flux and promotes carcinogenesis. Immunohistochemical analysis of esophageal tumors demonstrated reduced COX4 expression in the hypoxic core of the tumors, suggesting that a defect in the COX complex could contribute to cancer progression [23].

Together these observations indicate that cancer type has to be taken into consideration when analyzing COX4 expression. Moreover, functional studies have to be performed in order to determine the potential utility of COX4 as a biomarker of cancer progression or cancer cell response to treatment.

To clarify the functional importance of COX4 for thyroid cancer cells, we performed silencing experiments using DTC-derived (FTC133 and BCPAP) and MTC-derived (TT) cell lines. The level of COX4 expression was significantly decreased in all examined cells after transduction with shCOX4 lentiviral particles. However, the consequences of COX4 downregulation were different in DTC and MTC cells. Inhibition of COX4 had no significant effects on mitochondrial activity and activation of signaling pathways in FTC133 and BCPAP cells. In contrast, downregulation of COX4 in MTC-derived TT cells inhibited mitochondrial function and led to inhibition of p70S6K/pS6 and of p-ERK in MTC-derived TT cells. These results demonstrated that COX4 is involved in the maintenance of energy homeostasis in MTC-derived TT cells and suggest a role for alternative metabolic signaling in FTC- and PTC derived cell lines.

These cell type specific responses to COX4 inhibition could be explained by different abilities of DTC-derived and MTC-derived thyroid cancer cells to activate retrograde mitochondrial signaling. Mitochondria continuously communicate with the nucleus through ‘mitochondrial retrograde signaling’ to reconfigure cellular metabolism in response to mitochondrial stress [2,18]. Activation of glycolysis and increased utilization of glucose is one of the mechanisms by which cancer cells adapt to altered mitochondrial function. It has been recently shown that genetic silencing of COX4 by shRNA resulted in a metabolic shift to glycolysis [23].

It is possible that inhibition of COX4 resulted in a compensatory upregulation of a glucose transporter and an increased rate of glucose consumption, reminiscent of the Warburg effect in DTC-derived cells but not in MTC-derived TT cells [19]. Both FTC133 and BCPAP thyroid cancer cell lines have a “glycolytic” phenotype and are characterized by an increased expression of glycolytic genes when compared to normal thyroid [11,24,25]. In contrast, MTC-derived TT cells were characterized by a “mitochondrial” phenotype at baseline conditions and, in our experiments, these cells were unable to overcome the metabolic stress induced by COX4 knockdown. Another potential explanation for the difference we observed could be the different origin of DTC and MTC, with the neural crest origin of MTCs potentially being one of the factors underlying dependency of TT cells on mitochondrial respiration and specifically on COX activity.

The cytochrome c oxidase complex is known as a major regulator of mitochondrial reactive oxygen species (ROS) homeostasis [26]. It has been shown that deficiency in COX4-1 reduces COX activity and mitochondrial function and enhances the accumulation of cellular and mitochondrial ROS [27]. In our study, analysis of cancer-associated genes in COX4-expressing and COX4-deficient TT cells revealed upregulation of genes implicated in cellular response to DNA damage. These data were confirmed by Western blot analysis demonstrating an increase in γH2AX in shCOX4 TT cells, indicating activation of a DNA-repair signaling pathway. In addition, COX4 silencing led to induction of apoptosis in TT cells, as demonstrated by induction of caspase 3 cleavage.

We also examined the response of TT cells to treatment with a pharmacological inhibitor of COX activity—potassium cyanide (KCN). The effects of KCN on TT cells recapitulated data obtained in COX4-deficient TT cells (inhibition of mitochondrial function, induction of DNA damage and apoptosis). Together these data suggest that COX4 plays an important role in maintenance of ROS homeostasis in TT cells, and that downregulation of COX4 expression using either shRNA or pharmacological inhibition of COX activity leads to ROS-mediated induction of apoptosis.

Recent studies have identified mitochondria as a potential target for drug design and therapy [2,14,28,29,30]. Of particular interest is the use of antiparasitic drugs such as atovaquone that inhibits the cytochrome oxidase complex, or the antimicrobial tigecycline, which inhibits mitochondrial protein translation [31]. The availability of FDA-approved agents with an established side effect profile makes the potential repositioning in cancer an attractive option. Whether COX4 could represent a molecular target for treatment with these mitochondrial-active drugs needs to be studied and determined. The current study presents preliminary results demonstrating that COX4 could be a potential target agent for patients with MTC, but further in vivo studies are required.

## 4. Materials and Methods

### 4.1. Human Thyroid Tissue Samples

We conducted a retrospective analysis of thyroid tumor samples from patients operated on for benign and malignant thyroid nodules. Thyroid tissue samples were obtained from the Department of Pathology of the MedStar Washington Hospital Center, and from an archival thyroid tissue bank maintained at the Department of Pediatrics of the Uniformed Services University of the Health Sciences. The cases of this series consisted of 85 routinely processed formalin fixed paraffin embedded blocks from thyroid tumors operated in 1990–2000. Thyroid tissue samples were sectioned and histological diagnoses were established according to the WHO classification after examination of hematoxylin and eosin stained slides. There were 14 follicular adenomas (FA), 20 papillary thyroid carcinomas (PC) and 65 medullary carcinomas (MC), and we also examined 38 samples from normal thyroid tissue that was adjacent to the neoplasms. Furthermore, we used commercially available paraffin-embedded thyroid tissue microarray slides (US Biomax, Inc., Derwood, MD, USA). The microarrays included thyroid tissue samples from 26 follicular thyroid cancer (FTC), 56 papillary thyroid cancer (PC) and 10 samples from normal thyroid tissue. In total, we examined 48 samples from normal thyroid, 14 FA, 76 PTC, 26 FC and 65 MC.

A subset of pathology tissue samples (20 PC and 41 MTC) from the above group obtained from MedStar Washington Hospital Center and the Department of Pediatrics of the Uniformed Services University of the Health Sciences was subjected to Ion Torrent™ Oncomine™ Comprehensive Assay v3 (OCAv3) next-generation sequencing, analyzing single nucleotide variants (SNV), small insertions and deletions (INDEL), copy number variants (CNV) and gene fusions (GF) from 161 cancer driver genes.

The protocol was approved by the Institutional Review Boards of the MedStar Washington Hospital Center IRB ID: STUDY00001069.

### 4.2. Thyroid Cancer Cell Lines and Reagents

Human thyroid cancer cell lines (FTC133, FTC236, FTC238, BCPAP, and TT) were obtained from Dr. Motoyasu Saji (The Ohio State University) with permission from the researchers who originally established the cell lines. All thyroid cancer cell lines had been tested and authenticated by short tandem repeat profiling analysis to be of thyroid origin. These cell lines express common thyroid oncogenes, including *BRAFV600E* (BCPAP), loss of PTEN expression (FTC133, FTC236, FTC238), and *RETC634W* mutation in TT cells.

Cancer cells were propagated in conventional RPMI 1640 medium (Invitrogen, Carlsbad, CA, USA) supplemented with 10% fetal bovine serum (FBS), 100 U/mL penicillin, and 100 mg/mL streptomycin in a humidified 5% CO_2_ incubator. The cells were sub-cultured with 0.5% trypsin and 0.02% EDTA (Sigma–Aldrich, St. Louis, MO, USA) when the cell confluency reached 80%. All experiments were performed using thyroid cancer cell lines that had been passaged fewer than 20 times. Potassium cyanide (KCN) was purchased from Sigma–Aldrich (Sigma–Aldrich, St. Louis, MO, USA).

### 4.3. Depletion of the Endogenously Expressed COX4 in Thyroid Cancer Cell Lines

To establish cell lines in which COX4 expression is stably knocked down, FTC-derived (FTC133), PTC-derived (BCPAP) and MTC-derived (TT cells) cells were infected with lentiviral particles containing human COX4 specific or shRNA gene silencer sequences according to the manufacturer’s instructions (Santa Cruz Biotechnology, Santa Cruz, CA, USA). Control cell lines were infected with scrambled shRNA lentiviral particles that did not target any known mammalian mRNA, and contained a copGFP coding construct. Following transduction, cells were selected with puromycin (Sigma–Aldrich, St. Louis, MO, USA).

### 4.4. JC-1 Mitochondrial Membrane Potential Assay

Evaluation of mitochondrial membrane potential was performed with a fluorogenic lipophilic cation (JC-1; Cayman Chemical Company, Ann Arbor, MI, USA). In cells with hyperpolarized mitochondrial membranes, JC-1 spontaneously forms complexes (J-aggregates) with intense red fluorescence. In cells with depolarized mitochondrial membranes, JC-1 remains in the monomeric forms, which do not show red fluorescence. Detection of mitochondrial membrane potential was performed according to the manufacturer’s instructions followed by fluorescent microscopy. All experiments were repeated at least three times.

### 4.5. Mitochondrial Stress Assay

Mitochondrial function was determined by measuring oxygen consumption rate (OCR) of each cell line using XF Cell Mito Stress Test Kit (Agilent Technologies, Santa Clara, CA, USA). FTC133, BCPAP and TT cells transfected with shCOX4 and control cells were seeded in an XF96 cell culture microplate. The sensor cartridge and base medium were prepared by adding 1 mM pyruvate, 2 mM glutamine and 10 mM glucose and stored as per the manufacturer’s instructions. Seahorse assay was run in XF96 Extracellular Flux Analyzer (Agilent Technologies).

Following three baseline OCR measurements, cells were exposed sequentially to oligomycin (0.5 μM), carbonyl cyanide-4 (trifluoromethoxy) phenylhydrazone (FCCP) (1 μM), and rotenone/antimycin A (0.5 μM). Oligomycin inhibits ATP synthase (complex V), and the decrease in OCR following injection of oligomycin correlates to the mitochondrial respiration associated with cellular ATP production. FCCP is an uncoupling agent that collapses the proton gradient and disrupts the mitochondrial membrane potential, allowing cells to achieve maximal oxygen consumption rate. As a result, electron flow through the ETC (electron transfer chain) is uninhibited, and oxygen is maximally consumed by complex IV. The FCCP-stimulated OCR can then be used to calculate spare respiratory capacity, defined as the difference between maximal respiration and basal respiration. Spare respiratory capacity is a measure of the ability of the cell to respond to increased energy demand. The third injection is a mix of rotenone, a complex I inhibitor, and antimycin A, a complex III inhibitor. This combination shuts down mitochondrial respiration and enables the calculation of nonmitochondrial respiration driven by processes outside the mitochondria.

Three measurements were recorded after every injection. To normalize the results, cell number/well was quantified using the Celígo Imaging Cytometer (Nexcelom, Lawrence, MA, USA). The assay results were analyzed using Wave program 2.3.0 (Seahorse Bioscience, Billerica, MA, USA).

### 4.6. Protein Extraction and Western Blot Analysis

Thyroid cancer cells were incubated with ice-cold cell lysis buffer, scraped and centrifuged, and the supernatant was stored at −80 °C. Twenty-five micrograms of total protein lysate were suspended in reduced SDS sample buffer and the lysates were subjected to SDS–PAGE (4–12%). The separated proteins were transferred to a nitrocellulose membrane (Invitrogen, Life Technologies Corp., Carlsbad, CA, USA) by electrophoretic blotting.

Membranes were incubated overnight with primary antibody against β-actin (Sigma–Aldrich), p-AKT1/2/3 (Ser473), total AKT, p-ERK1/2, total ERK, cleaved caspase 3, phospho-p70S6K (Thr389), phospho-pS6 (Ser235/236), phospho-AMPK (Thr172), total AMPK, cytochrome c oxidase subunit 4 (COX4) (Cell Signaling Technology, Danvers, MA, USA), γH2AX, cyclin D1 and β-actin (Santa Cruz Biotechnology, Santa Cruz, CA, USA).

Detection of proteins was performed using the Li-Cor Odyssey imaging system (LI-COR Biosciences, Lincoln, NE, USA). Image Studio Lite version 3.1 (Licor) was used for band densitometry measurement and quantification of protein levels. 

### 4.7. RNA Extraction and Quantitative Real-Time PCR

Total RNA was extracted using the Allprep DNA/RNA mini kit (Qiagen, Germantown, MD, USA) according to the manufacturer’s protocol. The quality and quantity of total RNA was assessed with a Nanodrop 1000 spectrophotometer (Thermo Scientific, Wilimington, DE, USA). RNA (1 μg) was reverse transcribed into cDNA with the miScript II RT kit as indicated. SYBR green-based qPCR master mixes were obtained from Qiagen, Germantown, MD, USA. Real–time quantitative PCR screening of cancer associated genes was performed using a pre-fabricated RT-PCR Cancer Array (Qiagen, Germantown, MD, USA). The arrays were run on the Quantstudio Flex-6 (Thermofisher, Waltham, MA, USA). Cycle thresholds were determined for each gene using the instrument’s software. Cycle threshold values were transferred into a Data Analysis Template Excel file and ΔΔCt values determined for each gene. Fold changes in expression of each gene in experimental samples were compared to control.

Expression of cytochrome C oxidase 4 (COX4, with specific isoforms being COX4-1 and COX4-2) were examined using commercially available primers (Qiagen, Germantown, MD, USA). The PCR reactions were performed in triplicate on a Lightcycler 96 (Roche Diagnostics, Indiananpolis, IN, USA) with amplification profile of the hold stage at 50 °C for 2 min, followed by 40 cycles of denaturation for 10 min at 95 °C, and annealing for 1 min at 60 °C. Melting curves were determined to ensure amplification of a single product. Negative cDNA controls were cycled in parallel with each run. The number of transcripts were normalized to that of 18S. Baseline and threshold values were set by the Lightcycler software and data was analyzed and expressed using the 2-Delta CT method of relative quantification.

### 4.8. Immunostaining

Immunostaining was performed on thyroid tissue microarray slides and on thyroid tumor sections. Formalin-fixed paraffin-embedded tissue sections were dewaxed in xylene, soaked in alcohol, and after microwave treatment in antigen unmasking solution (Vector Labs, Burlingame, CA, USA) for 10 min, endogenous peroxidase activity was quenched by incubation in 3% hydrogen peroxide. Sections were then incubated for 10 min in working solution of blocking serum, and incubated at 4 °C overnight with anti-COX4 (2 μg/mL) (Santa Cruz Biotechnology, Santa Cruz, CA, USA). Immunostaining was performed using the Vectastain Universal Quick Kit according to the manufacturer’s instructions. Sections were incubated with biotinylated secondary antibody for 10 min, and in streptavidin/peroxidase complex working solution for 10 min. Peroxidase staining was revealed with ImmPact DAB, peroxidase substrate Kit (Vector Labs, Burlingame, CA, USA). Sections were counterstained with hematoxylin and mounted. Antiserum was omitted in the negative control.

The intensity and extent of COX IV immunostaining were evaluated for all samples under double-blinded conditions. Samples demonstrating moderate or strong staining in more than 50% of cells were considered as positive.

### 4.9. Statistical Analysis

Data were summarized by using percentages for categorical variables and means ± standard deviation (SD) or medians with inter quartile range (IQR) for continuous variables. Categorical variables were compared between subgroups with Pearson Chi-square test. Continuous variables were compared with Student’s *t*-test and with the Mann–Whitney test when appropriate. *p* < 0.05 was considered to indicate a statistically significant result. The statistical analyses were performed using the SPSS 24.0 software.

## 5. Conclusions

In conclusion, these observations indicate that there is significant heterogeneity in the metabolic requirements among thyroid cancer cells, and that development of thyroid cancer, especially MTC, is associated with increased expression of COX4. Results of in vitro experiments indicated that COX4 is implicated in the regulation of energy homeostasis in MTC-derived cells and suggested that evaluation of mitochondrial metabolic markers in tumor samples could prove useful for selection of therapeutic strategies in patients with medullary thyroid cancer.

## Figures and Tables

**Table 1:** Analysis of immunostaining with anti-COX4 in function of histology.

Histology	COX4 Immunostaining		Statistical Comparison
	Negative	Positive	*p* value
			(vs. normal tissue)
Normal tissue	47 (97.9%)	1 (2.1%)	
Follicular adenomas	13 (92.9%)	1 (7.1%)	0.346
Follicular thyroid cancer	15 (57.7%)	11 (42.3%)	0.0001
Papillary thyroid cancer	34 (44.7%)	42 (55.3%)	0.0001

**Table 2:** Analysis of COX4 expression in function of clinicopathological features in patients with medullary thyroid carcinomas.

Clinicopathological Features	COX4 Immunostaining		*p* Value
	negative	positive	
Age	52.3 ± 13.8	52.9 ± 15.7	0.89
Size (cm)	1.5 (1–2)	2 (1.2–3.8)	0.04
Gender			
female	8 (20.5%)	31 (79.5%)	0.49
male	7 (28%)	18 (72%)	
Lymph node metastasis			0.024
No	13 (35.1%)	24 (64.9%)	
Yes	3 (10.7%)	25 (89.3%)	
Mutation status			
No detectable mutations	7 (53.8%)	6 (46.2%)	0.014
*RET*	2 (9.1%)	20 (90.9%)	
*RAS*	2 (33.3%)	4 (66.7%)	

## Supplementary Materials

The following are available online at https://www.mdpi.com/2072-6694/12/9/2548/s1, Table S1: Real Time PCR screening of genes with comparison between shCOX4 and scamble.

## Disclaimer

The views expressed in this article are those of the authors and do not reflect the official policy or position of the United States Air Force, the Department of Defense, or the U.S. government. Title 17 U.S.C. 105 provides that “copyright protection under this title is not available for any work of the United States Government.” Title 17 U.S.C. 101 defines a United States government work as “a work prepared by a military service member or employee of the United States government as part of that person’s official duties.” This work was prepared as part of the official duties of Jensen, Vasko, Reynolds, Mendonca-Torres, Costello, Patel.

## References

[1] Buchakjian M.R., Kornbluth S. (2010). The engine driving the ship: Metabolic steering of cell proliferation and death. Nat. Rev. Mol. Cell Boil..

[2] Wallace D.C. (2012). Mitochondria and cancer. Nat. Rev. Cancer.

[3] Ishikawa K., Takenaga K., Akimoto M., Koshikawa N., Yamaguchi A., Imanishi H., Nakada K., Honma Y., Hayashi J.-I. (2008). ROS-Generating Mitochondrial DNA Mutations Can Regulate Tumor Cell Metastasis. Science.

[4] Ordys B.B., Launay S., Deighton R.F., McCulloch J., Whittle I.R. (2010). The Role of Mitochondria in Glioma Pathophysiology. Mol. Neurobiol..

[5] Grupp K., Jedrzejewska K., Tsourlakis M.C., Koop C., Wilczak W., Adam M., Quaas A., Sauter G., Simon R., Izbicki J.R. (2013). High mitochondria content is associated with prostate cancer disease progression. Mol. Cancer.

[6] Santidrian A.F., Matsuno-Yagi A., Ritland M., Seo B.B., Leboeuf S., Gay L.J., Yagi T., Felding-Habermann B. (2013). Mitochondrial complex I activity and NAD+/NADH balance regulate breast cancer progression. J. Clin. Investig..

[7] Kadenbach B., Hüttemann M., Arnold S., Lee I., Bender E. (2000). Mitochondrial energy metabolism is regulated via nuclear-coded subunits of cytochrome c oxidase. Free Radic. Boil. Med..

[8] Napiwotzki J., Kadenbach B. (1998). Extramitochondrial ATP/ADP-Ratios Regulate Cytochrome c Oxidase Activity via Binding to the Cytosolic Domain of Subunit IV. Boil. Chem..

[9] Fukuda R., Zhang H., Kim J.-W., Shimoda L., Dang C.V., Semenza G.L. (2007). HIF-1 Regulates Cytochrome Oxidase Subunits to Optimize Efficiency of Respiration in Hypoxic Cells. Cell.

[10] Arnold S., Kadenbach B. (1999). The intramitochondrial ATP/ADP-ratio controls cytochrome c oxidase activity allosterically. FEBS Lett..

[11] Bikas A., Jensen K., Patel A., Costello J., McDaniel D., Klubo-Gwiezdzinska J., Larin A., Hoperia V., Burman K.D., Boyle L. (2015). Glucose-deprivation increases thyroid cancer cells sensitivity to metformin. Endocr. Relat. Cancer.

[12] Klubo-Gwiezdzinska J., Costello J., Patel A., Bauer A., Jensen K., Mete M., Burman K.D., Wartofsky L., Vasko V. (2013). Treatment With Metformin Is Associated With Higher Remission Rate in Diabetic Patients With Thyroid Cancer. J. Clin. Endocrinol. Metab..

[13] Thakur S., Daley B., Gaskins K., Vasko V.V., Boufraqech M., Patel D., Sourbier C., Reece J.M., Cheng S.-Y., Kebebew E. (2018). Metformin Targets Mitochondrial Glycerophosphate Dehydrogenase to Control Rate of Oxidative Phosphorylation and Growth of Thyroid Cancer In Vitro and In Vivo. Clin. Cancer Res..

[14] Klubo-Gwiezdzinska J., Jensen K., Costello J., Patel A., Hoperia V., Bauer A., Burman K.D., Wartofsky L., Vasko V. (2012). Metformin inhibits growth and decreases resistance to anoikis in medullary thyroid cancer cells. Endocr. Relat. Cancer.

[15] Starenki D., Park J.-I. (2013). Mitochondria-targeted nitroxide, Mito-CP, suppresses medullary thyroid carcinoma cell survival in vitro and in vivo. J. Clin. Endocrinol. Metab..

[16] Alvero A.B., Montagna M.K., Holmberg J.C., Craveiro V., Brown D., Mor G. (2011). Targeting the mitochondria activates two independent cell death pathways in ovarian cancer stem cells. Mol. Cancer Ther..

[17] Payen V.L., Porporato P.E., Baselet B., Sonveaux P. (2015). Metabolic changes associated with tumor metastasis, part 1: Tumor pH, glycolysis and the pentose phosphate pathway. Cell. Mol. Life Sci..

[18] Ward P.S., Thompson C.B. (2012). Signaling in Control of Cell Growth and Metabolism. Cold Spring Harb. Perspect. Boil..

[19] Koppenol W.H., Bounds P.L., Dang C.V. (2011). Otto Warburg’s contributions to current concepts of cancer metabolism. Nat. Rev. Cancer.

[20] Campian J.L., Gao X., Qian M., Eaton J.W. (2007). CytochromecOxidase Activity and Oxygen Tolerance. J. Boil. Chem..

[21] Griguer C.E., Cantor A.B., Fathallah-Shaykh H.M., Gillespie G.Y., Gordon A.S., Markert J.M., Radovanovic I., Clement-Schatlo V., Shannon C.N., Oliva C.R. (2013). Prognostic Relevance of Cytochrome c Oxidase in Primary Glioblastoma Multiforme. PLoS ONE.

[22] Whitaker-Menezes D., Martinez-Outschoorn U., Flomenberg N., Birbe R.C., Witkiewicz A.K., Howell A., Pavlides S., Tsirigos A., Ertel A., Pestell R.G. (2011). Hyperactivation of oxidative mitochondrial metabolism in epithelial cancer cells in situ: Visualizing the therapeutic effects of metformin in tumor tissue. Cell Cycle.

[23] Srinivasan S., Guha M., Dong D.W., Whelan K.A., Ruthel G., Uchikado Y., Natsugoe S., Nakagawa H., Avadhani N.G. (2015). Disruption of cytochrome c oxidase function induces the Warburg effect and metabolic reprogramming. Oncogene.

[24] Morani F., Phadngam S., Follo C., Titone R., Thongrakard V., Galetto A., Alabiso O., Isidoro C. (2014). PTEN deficiency and mutant p53 confer glucose-addiction to thyroid cancer cells: Impact of glucose depletion on cell proliferation, cell survival, autophagy and cell migration. Genes Cancer.

[25] Morani F., Phadngam S., Follo C., Titone R., Aimaretti G., Galetto A., Alabiso O., Isidoro C. (2014). PTEN regulates plasma membrane expression of glucose transporter 1 and glucose uptake in thyroid cancer cells. J. Mol. Endocrinol..

[26] Sena L.A., Chandel N.S. (2012). Physiological Roles of Mitochondrial Reactive Oxygen Species. Mol. Cell.

[27] Oliva C.R., Markert T., Gillespie G.Y., Griguer C.E. (2015). Nuclear-encoded cytochrome c oxidase subunit 4 regulates BMI1 expression and determines proliferative capacity of high-grade gliomas. Oncotarget.

[28] Ubah O.C., Wallace H.M. (2014). Cancer therapy: Targeting mitochondria and other sub-cellular organelles. Curr. Pharm. Des..

[29] Han B., Cui H., Kang L., Zhang X., Jin Z., Lu L., Fan Z. (2015). Metformin inhibits thyroid cancer cell growth, migration, and EMT through the mTOR pathway. Tumor Boil..

[30] Kim H., Yang J., Kim M.J., Choi S., Chung J.-R., Kim J.-M., Yoo Y.H., Chung J., Koh H. (2015). Tumor Necrosis Factor Receptor-associated Protein 1 (TRAP1) Mutation and TRAP1 Inhibitor Gamitrinib-triphenylphosphonium (G-TPP) Induce a Forkhead Box O (FOXO)-dependent Cell Protective Signal from Mitochondria. J. Boil. Chem..

[31] Haq R., Fisher D.E., Widlund H.R. (2014). Molecular pathways: BRAF induces bioenergetic adaptation by attenuating oxidative phosphorylation. Clin. Cancer Res..

