# Supplementary material for: Cytochrome C Oxidase Subunit 4 (COX4): A Potential Therapeutic Target for the Treatment of Medullary Thyroid Cancer
Source: Cancers (Basel). 2020 Sep 8;12(9):2548. doi: 10.3390/cancers12092548 (PMC7565757; doi:10.3390/cancers12092548)
Supplement: Supplementary file 1 [file cancers-12-02548-s001.pdf]

cancers

MDPI

Article

Cytochrome C Oxidase Subunit 4 (COX4): A Potential Therapeutic Target for the Treatment of Medullary Thyroid Cancer

Athanasios Bikas, Kirk Jensen, Aneeta Patel, John Costello, Sarah M. Reynolds, Maria Cecilia Mendonca-Torres, Shilpa Thakur, Joanna Klubo-Gwiezdzinska, Dorina Ylli, Leonard Wartofsky, Kenneth Burman and Vasyl Vasko

Supplementary Materials:

Table S1. Real Time PCR screening of genes with comparison between shCOX4 and scamble.

Position	Symbol	Description	Fold changes in shCOX4 vs scramble		
			FTC133	BCPAP	TT
1	ACLY	ATP citrate lyase	1.40	-1.01	-2.05
2	ACSL4	Acyl-CoA synthetase long-chain family member 4	1.13	1.36	1.01
3	ADM	Adrenomedullin	-1.04	-2.62	1.82
4	ANGPT1	Angiopoietin 1	-2.11	1.36	-1.43
5	ANGPT2	Angiopoietin 2	**-3.39**	**-4.84**	**-4.74**
6	APAF1	Apoptotic peptidase activating factor 1	1.32	-1.21	1.12
7	ARNT	Aryl hydrocarbon receptor nuclear translocator	-1.28	1.25	1.45
8	ATP5A1	ATP synthase, H+ transporting, mitochondrial F1 complex, alpha subunit 1, cardiac muscle	1.36	1.37	-1.07
9	AURKA	Aurora kinase A	1.42	-1.27	-1.89
10	BCL2L11	BCL2-like 11 (apoptosis facilitator)	-1.64	1.59	-1.00
11	BIRC3	Baculoviral IAP repeat containing 3	**-3.76**	**3.06**	1.77
12	BMI1	BMI1 polycomb ring finger oncogene	-1.02	1.00	-1.63
13	CA9	Carbonic anhydrase IX	-1.31	**-17.94**	**18.30**
14	CASP2	Caspase 2, apoptosis-related cysteine peptidase	-1.13	1.73	-2.72
15	CASP7	Caspase 7, apoptosis-related cysteine peptidase	1.03	1.07	2.14
16	CASP9	Caspase 9, apoptosis-related cysteine peptidase	-1.78	-1.08	-1.51
17	CCL2	Chemokine (C-C motif) ligand 2	-2.68	1.03	2.24
18	CCND2	Cyclin D2	-2.68	1.03	2.24
19	CCND3	Cyclin D3	-1.12	1.37	-2.06
20	CDC20	Cell division cycle 20 homolog (S. cerevisiae)	-1.09	1.04	-1.35
21	CDH2	Cadherin 2, type 1, N-cadherin (neuronal)	1.60	1.04	-1.52
22	CFLAR	CASP8 and FADD-like apoptosis regulator	1.31	1.25	-1.53
23	COX5A	Cytochrome c oxidase subunit Va	1.16	-1.11	1.07
24	CPT2	Carnitine palmitoyltransferase 2	**-4.25**	1.45	-1.32
25	DDB2	Damage-specific DNA binding protein 2, 48kDa	-2.17	-1.21	-1.13
26	DDIT3	DNA-damage-inducible transcript 3	**-5.65**	1.08	**3.79**
27	DKC1	Dyskeratosis congenita 1, dyskerin	1.38	1.77	-2.35
28	DSP	Desmoplakin	**-7.26**	1.01	1.09
29	E2F4	E2F transcription factor 4, p107/p130-binding	-1.10	1.22	**-3.47**
30	EPO	Erythropoietin	2.95	1.03	1.60
31	ERCC3	Excision repair cross-complementing rodent repair deficiency, complementation group 3 (xeroderma pigmentosum group B complementing)	-1.11	1.28	-2.36
32	ERCC5	Excision repair cross-complementing rodent repair deficiency, complementation group 5	-1.74	1.30	1.24
33	ETS2	V-Ets erythroblastosis virus E26 oncogene homolog 2 (avian)	-2.00	1.18	-1.34
34	FASLG	Fas ligand (TNF superfamily, member 6)	1.08	1.03	1.60
35	FGF2	Fibroblast growth factor 2 (basic)	-2.87	1.79	-1.27
36	FLT1	Fms-related tyrosine kinase 1 (vascular endothelial growth factor/vascular permeability factor receptor)	1.08	1.03	1.60
37	FOXC2	Forkhead box C2 (MFH-1, mesenchyme forkhead 1)	1.08	-1.98	1.60
38	G6PD	Glucose-6-phosphate dehydrogenase	-1.26	-1.27	-1.62
39	GADD45G	Growth arrest and DNA-damage-inducible, gamma	1.08	1.03	-2.17
40	GPD2	Glycerol-3-phosphate dehydrogenase 2 (mitochondrial)	-1.41	1.11	-2.57

41	GSC	Goosecoid homeobox	1.08	1.03	1.60
42	HMOX1	Heme oxygenase (decycling) 1	1.64	1.85	**4.01**
43	IGFBP3	Insulin-like growth factor binding protein 3	-1.32	-2.05	**8.69**
44	IGFBP5	Insulin-like growth factor binding protein 5	1.12	-2.74	**3.06**
45	IGFBP7	Insulin-like growth factor binding protein 7	2.05	-1.38	-1.52
46	KDR	Kinase insert domain receptor (a type III receptor tyrosine kinase)	1.41	1.14	**-32.12**
47	KRT14	Keratin 14	1.08	**-6.45**	-2.59
48	LDHA	Lactate dehydrogenase A	-1.29	1.11	1.85
49	LIG4	Ligase IV, DNA, ATP-dependent	-2.01	1.67	-2.34
50	LPL	Lipoprotein lipase	**-3.37**	1.76	-1.50
51	MAP2K1	Mitogen-activated protein kinase kinase 1	1.31	-1.11	-1.39
52	MAP2K3	Mitogen-activated protein kinase kinase 3	1.90	1.41	-1.47
53	MAPK14	Mitogen-activated protein kinase 14	1.32	-1.34	-1.25
54	MCM2	Minichromosome maintenance complex component 2	-1.45	1.91	**-3.49**
55	MKI67	Antigen identified by monoclonal antibody Ki-67	-1.01	-1.68	**-4.17**
56	NOL3	Nucleolar protein 3 (apoptosis repressor with CARD domain)	1.28	-1.10	2.34
57	OCLN	Occludin	-1.53	1.29	-1.61
58	PFKL	Phosphofructokinase, liver	-1.51	-1.13	-1.02
59	PGF	Placental growth factor	**-4.19**	1.08	1.60
60	PINX1	PIN2/TERF1 interacting, telomerase inhibitor 1	-1.44	**-8.95**	**-4.00**
61	POLB	Polymerase (DNA directed), beta	-1.72	-1.00	-2.27
62	PPP1R15A	Protein phosphatase 1, regulatory (inhibitor) subunit 15A	-1.82	1.60	2.97
63	SERPINB2	Serpin peptidase inhibitor, clade B (ovalbumin), member 2	1.08	1.24	1.48
64	SERPINF1	Serpin peptidase inhibitor, clade F (alpha-2 antiplasmin), member 1	**-5.21**	**3.01**	**3.34**
65	SKP2	S-phase kinase-associated protein 2 (p45)	2.17	1.58	**-3.98**
66	SLC2A1	Solute carrier family 2 (facilitated glucose transporter), member 1	1.37	-2.01	1.60
67	SNAI1	Snail homolog 1 (Drosophila)	-2.42	2.38	1.88
68	SNAI2	Snail homolog 2 (Drosophila)	-1.79	1.15	-2.12
69	SNAI3	Snail homolog 3 (Drosophila)	**3.50**	1.03	1.48
70	SOD1	Superoxide dismutase 1, soluble	-1.17	1.30	-1.44
71	SOX10	SRY (sex determining region Y)-box 10	1.08	1.03	1.60
72	STMN1	Stathmin 1	-1.04	-1.27	-2.43
73	TBX2	T-box 2	-1.42	1.03	2.91
74	TEK	TEK tyrosine kinase, endothelial	-1.00	1.03	1.60
75	TEP1	Telomerase-associated protein 1	1.45	1.34	-1.92
76	TERF1	Telomeric repeat binding factor (NIMA-interacting) 1	-1.00	1.23	-2.94
77	TERF2IP	Telomeric repeat binding factor 2, interacting protein	-1.21	1.62	-1.09
78	TINF2	TERF1 (TRF1)-interacting nuclear factor 2	-1.34	-1.33	1.19
79	TNKS	Tankyrase, TRF1-interacting ankyrin-related ADP-ribose polymerase	-2.13	1.56	-1.27
80	TNKS2	Tankyrase, TRF1-interacting ankyrin-related ADP-ribose polymerase 2	-1.51	1.29	-1.05
81	UQCRFS1	Ubiquinol-cytochrome c reductase, Rieske iron-sulfur polypeptide 1	-1.23	1.18	-1.16
82	VEGFC	Vascular endothelial growth factor C	-1.23	1.32	1.60
83	WEE1	WEE1 homolog (S. pombe)	-1.37	-1.02	**-3.08**
84	XIAP	X-linked inhibitor of apoptosis	-2.20	1.03	-1.95

Figure 1A

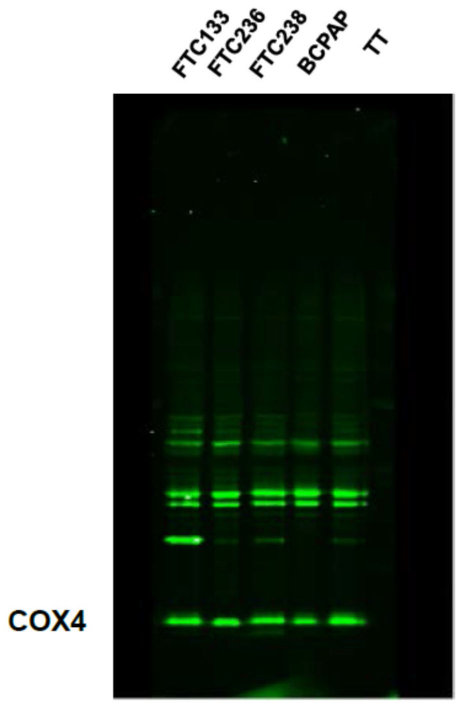

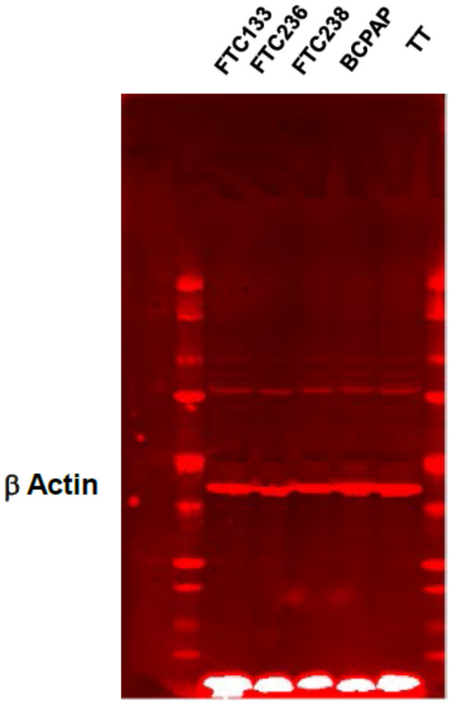

Figure 1C

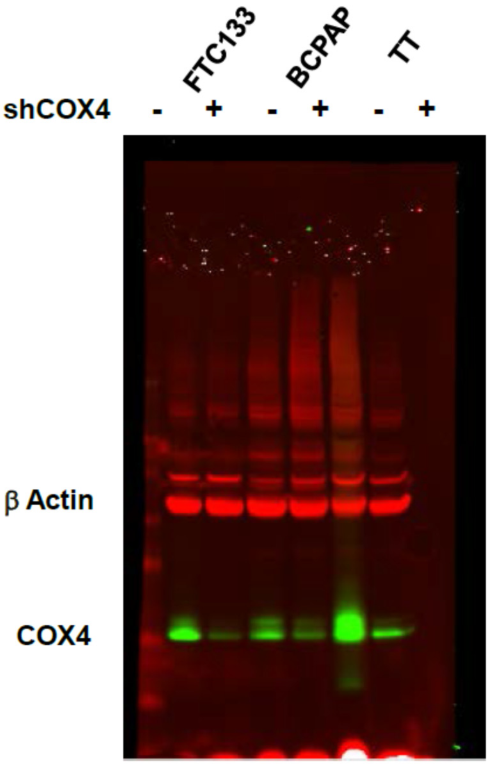

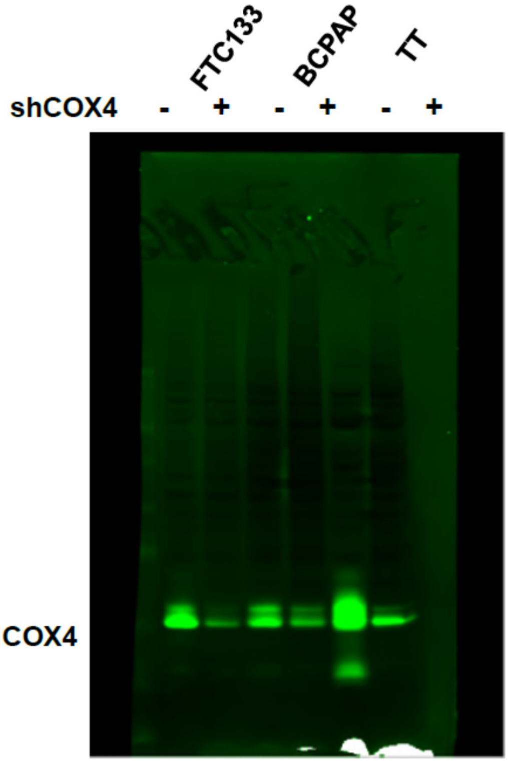

Figure 5A

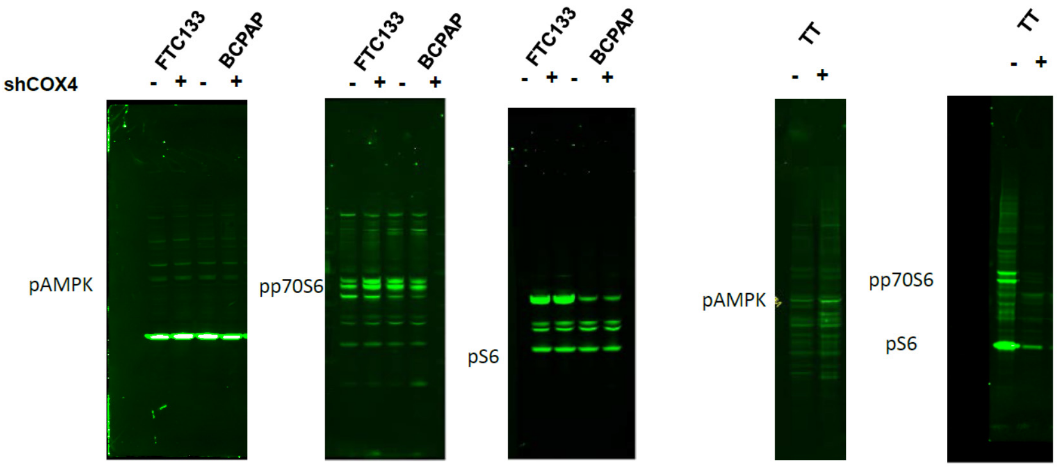

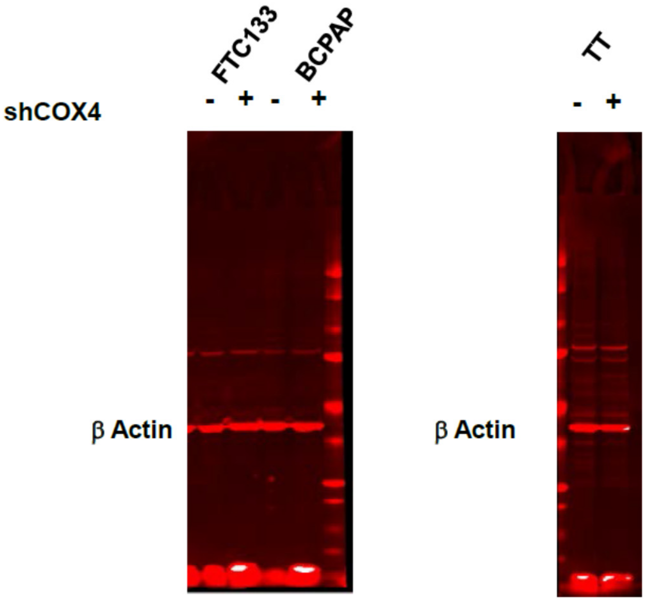

Figure 5B

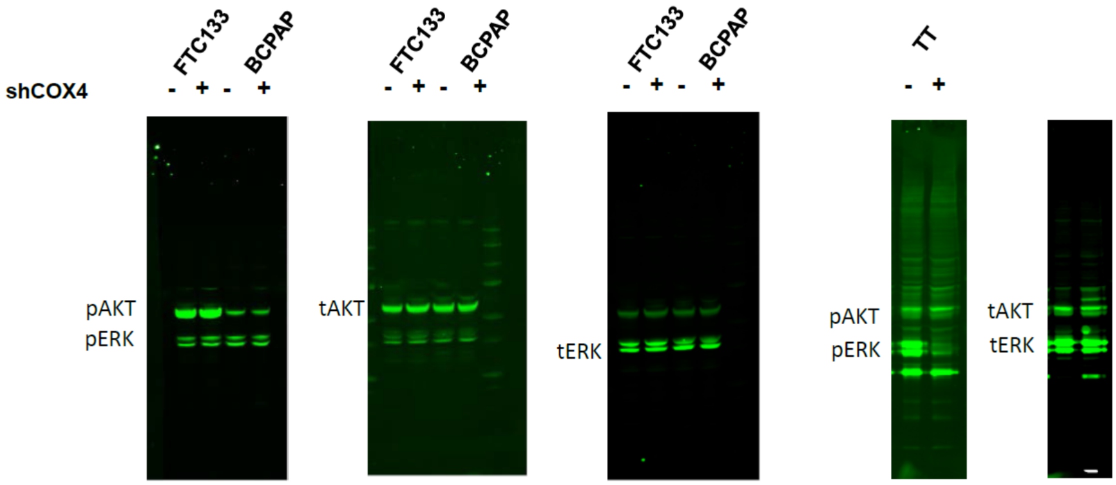

Figure 5C and D

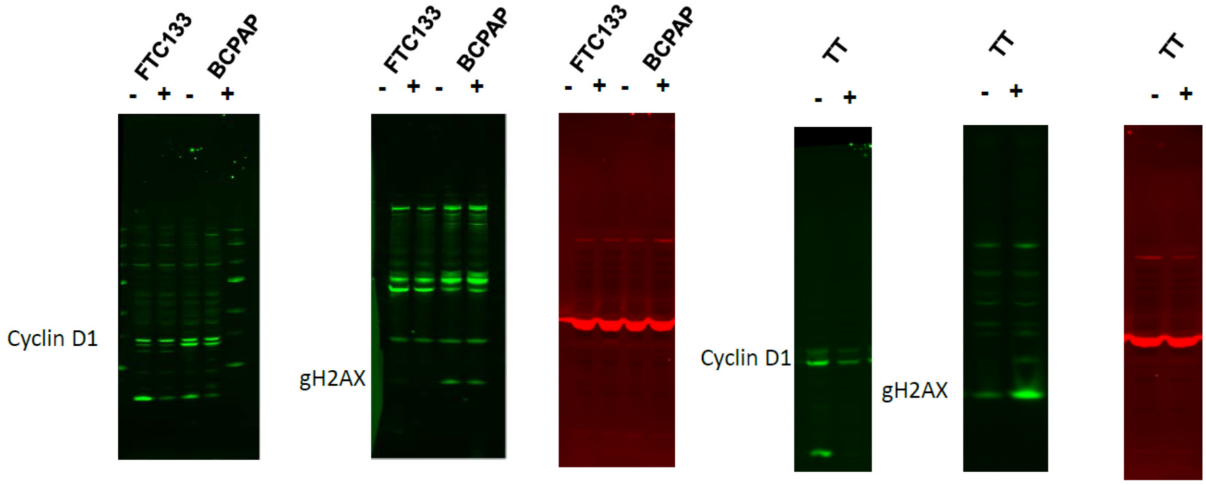

Figure 6E

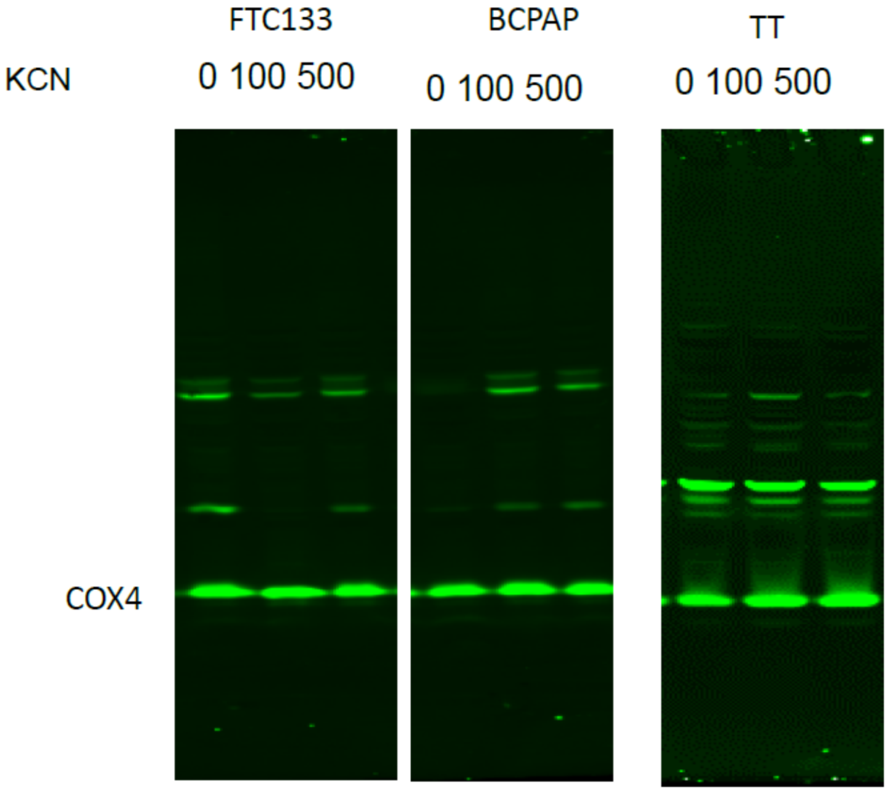

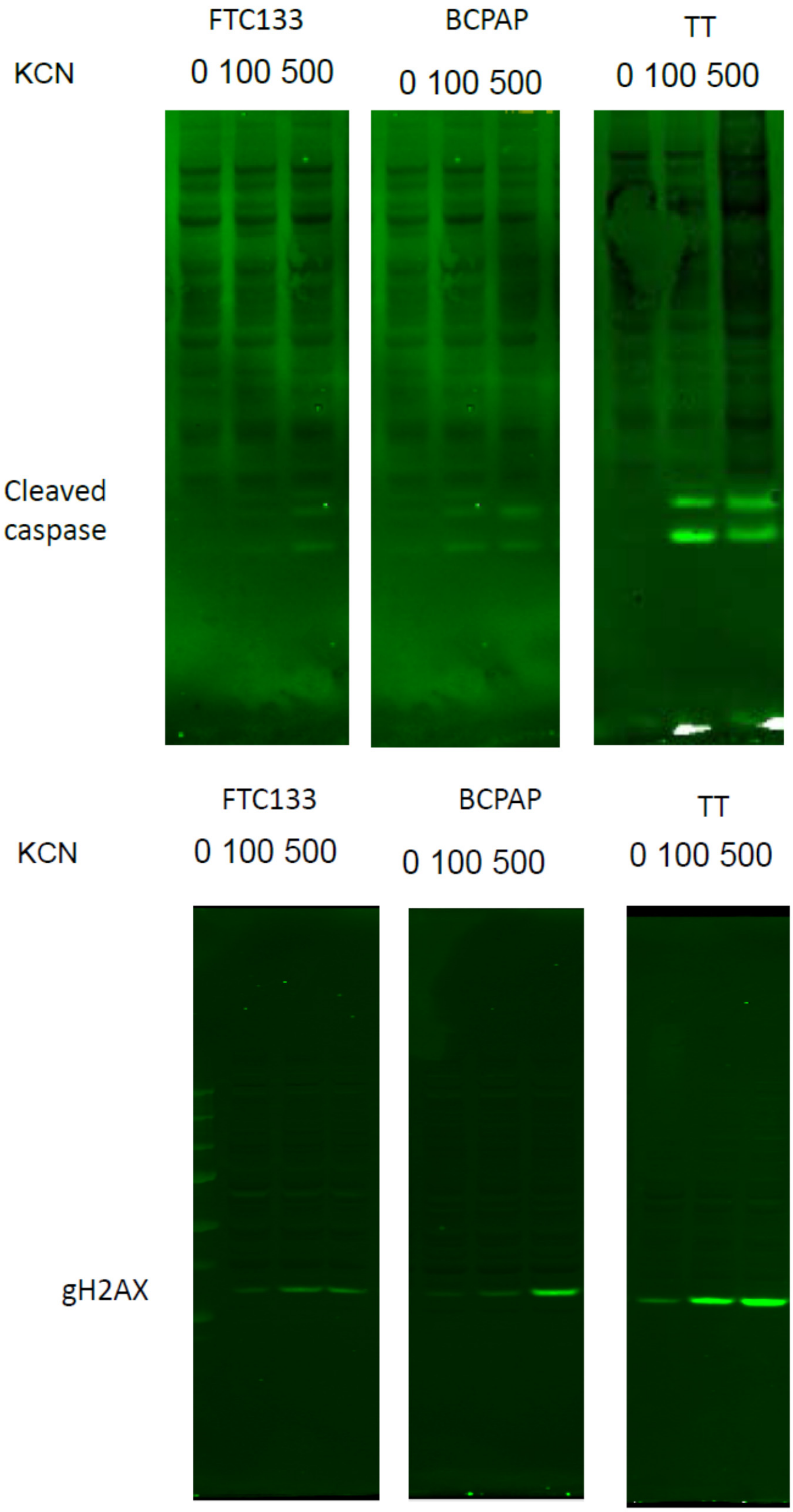

FTC133
BCPAP
TT
KCN
0 100 500
0 100 500
0 100 500
Cleaved caspase
FTC133
BCPAP
TT
KCN
0 100 500
0 100 500
0 100 500
gH2AX

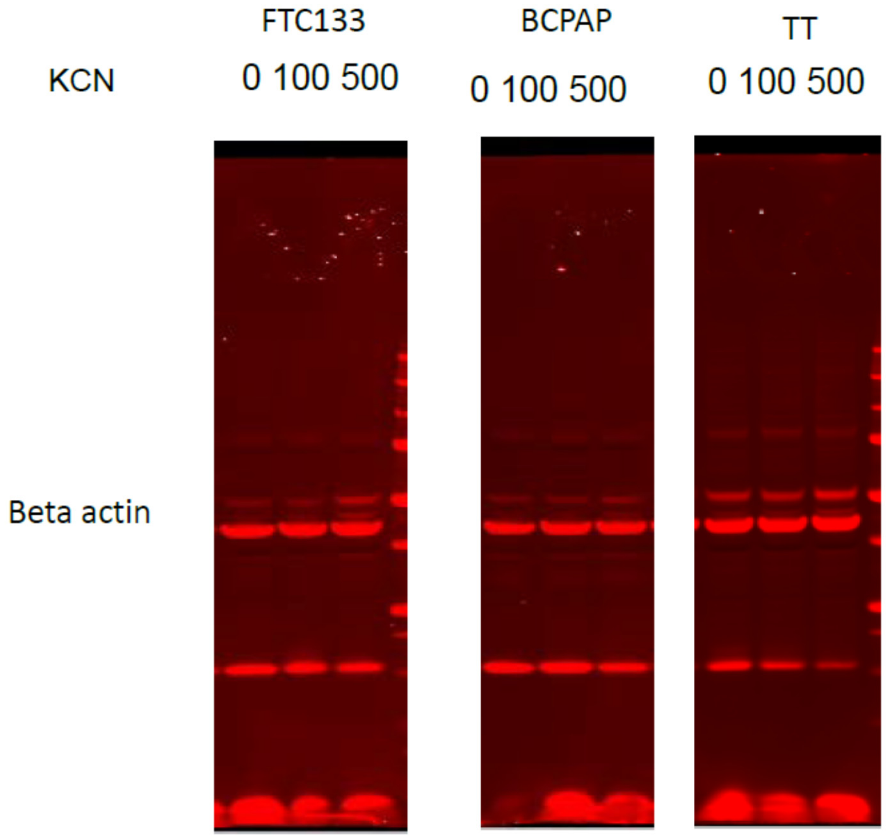

© 2020 by the authors. Licensee MDPI, Basel, Switzerland. This article is an open access article distributed under the terms and conditions of the Creative Commons Attribution (CC BY) license (http://creativecommons.org/licenses/by/4.0/).
